# Bidirectional association between disturbed sleep and neuropathic pain symptoms: a prospective cohort study in post-total joint replacement participants

**DOI:** 10.2147/JPR.S149830

**Published:** 2018-06-06

**Authors:** Joanne Stocks, Nicole KY Tang, David A Walsh, Sophie C Warner, Hollie L Harvey, Wendy Jenkins, Abhishek Abhishek, Michael Doherty, Ana M Valdes

**Affiliations:** 1 Academic Rheumatology, University of Nottingham, Nottingham, UK; 2 Arthritis Research UK Pain Centre, University of Nottingham, Nottingham, UK; 3 National Institute for Health Research, Nottingham Biomedical Research Centre, Nottingham, UK; 4 Department of Psychology, University of Warwick, Coventry, UK

**Keywords:** total joint replacement, opioids, osteoarthritis

## Abstract

**Background:**

Disturbed sleep is strongly correlated with chronic pain. The aim of this study was to examine the association between sleep disturbance and incident joint pain focusing on neuropathic-like pain symptoms.

**Methods:**

A total of 423 individuals who had undergone total joint replacement (TJR) for osteoarthritis were assessed at the mean time of 3.6 years post-surgery and again at 5.9 years post-TJR, using the Medical Outcomes Survey sleep subscale, Western Ontario and McMaster Universities Osteoarthritis Index (WOMAC), and painDETECT questionnaire instruments. Cox hazard ratios (HRs) and 95% confidence intervals (CIs) were computed adjusting for age, body mass index, sex, and use of hypnotic and analgesic medication.

**Results:**

The presence of neuropathic pain symptoms predicted incidence of disturbed sleep after adjustment for covariates and pain severity (adjusted HR [aHR] 2.01, 95% CI: 1.00–4.10; *p*<0.05). There was no association between joint pain and incidence of disturbed sleep when individuals with neuropathic pain symptoms at the baseline visit were excluded (aHR 1.11, 95% CI: 0.47–2.67). Disturbed sleep at baseline predicted incident neuropathic joint pain symptoms (aHR 2.75, 95% CI: 1.21–6.26; *p*<0.016) but had no effect on incidence of joint pain when all types of pain were considered together (aHR 0.63, 95% CI: 0.30–1.39).

**Conclusion:**

These data suggest a causal bidirectional link between sleep disturbance and joint pain with neuropathic features but not with other types of joint pain.

## Plain language summary

### Why was the study done?

People experiencing long-term pain commonly complain of disturbed and insufficient quality sleep, which is associated with high blood pressure, type 2 diabetes and obesity. Changes in the nervous system can cause neuropathic-like pain symptoms, and of 25–30% of people with painful osteoarthritis (OA), who have had a total joint replacement (TJR), 25–30% still report symptoms of neuropathic pain. This study aimed to discover a possible link between sleep and long-term joint pain and if neuropathic-like pain symptoms play a role.

### What did the researchers do and find?

A total of 423 people with OA, who had previously undergone TJR, were assessed. In these people, a strong link was found between long-term joint pain and disturbed sleep. This increased incidence of disturbed sleep linked specifically to neuropathic-like pain symptoms and not merely to the presence of pain. The researchers also found that disturbed sleep increases the risk of developing new long-term pain in previously pain-free individuals.

### What do these results mean?

In people with OA who have undergone TJR, neuropathic pain can predict poor sleep and vice versa. Improving sleep disturbance and joint pain has the potential to improve outcomes after joint replacement surgery, particularly if pain-relieving strategies target neuropathic joint pain.

## Introduction

Disturbed sleep is a frequent complaint of people experiencing chronic pain such as those with knee osteoarthritis (OA).^1^ The resultant changes in sleep architecture can affect health even in the presence of apparently adequate sleep duration. For example, an insufficient amount of slow-wave sleep associates with hypertension, type 2 diabetes mellitus and obesity.^2^ Sleep disturbances are present in 67–88% of people with chronic pain, and ≥50% of individuals with insomnia have chronic pain.^3^ Sleep and pain symptoms are considered to be reciprocally interacting.^4^ Sleep disturbance affects nociceptive responses in healthy, pain-free volunteers.^5^ Equally, pain affects sleep quality. For example, a prospective UK study^6^ and a recent study from Norway^7^ have shown that widespread pain predicts the incidence of insomnia over a 3-year period. Furthermore, good sleep improves the long-term prognosis of individuals with tension-type headache, migraine and chronic musculoskeletal pain.^4^,^6^

It has been suggested that the hyperalgesic effect of sleep deprivation is mediated primarily by impairments in the descending pain modulatory systems^4^,^8^ rather than by amplification of the ascending sensory pathways. A compromised pain inhibitory capacity has been demonstrated in many idiopathic clinical pain conditions with prominent sleep disturbance such as fibromyalgia.^9^

Total joint replacement (TJR) surgery is a cost-effective and safe intervention for severe large-joint OA,^10^ but the health-related quality of life after hip and knee arthroplasty is considerably lower than that reported by the general population in the same age range.^11^ More than 20% of individuals report moderate to marked persistent pain after 1 year of surgery,^12^ and a substantial proportion of these people also report neuropathic-like pain symptoms.^13^

Neuropathic-like pain symptoms are caused by changes or damage to the nervous system, which can result from chronic nociceptive input (as seen in chronic pain states) and nerve damage during surgery.^14^,^15^ Neuropathic pain has been reported in people with OA and post-TJR.^12^,^16^ Although the majority of people with OA do not report neuropathic pain,^17^,^18^ reflecting predominantly nociceptive mechanisms in this disease, a considerable proportion of them (20–35%) report this type of symptoms. Moreover, people with OA who report neuropathic pain symptoms are at a greater risk of poor outcome following TJR surgery,^19^ and those with persistent post-arthroplasty pain frequently describe neuropathic pain symptoms.^13^ However, the link between disturbed sleep and chronic joint pain, in particular neuropathic joint pain, has not been explored to date.

We hypothesized that sleep disturbance in individuals with chronic joint pain could be induced by neuropathic pain symptoms. In order to explore this, we have examined the data collected at two time points, 2 years apart, in a cohort of individuals who underwent knee or hip TJR for OA.^20^

Given the links between descending pain inhibition and sleep, we focused in particular on the links between disturbed sleep and neuropathic pain seeking to answer two study questions: 1) is the link between chronic joint pain and sleep mediated by the presence of neuropathic pain symptoms? and 2) does sleep disturbance predict the development of neuropathic pain symptoms over a 2-year period?

## Participants and methods

### Study participants

People who had undergone total hip or knee replacement surgery were identified from hospital orthopedic clinic lists in the Nottinghamshire area, UK, and were mailed an invitation to participate in the study. Approval for the study was obtained from the research ethics committees of Nottingham City Hospital, North Nottinghamshire, and all participants provided written informed consent. Consenting participants underwent a home visit by a research nurse between 2008 and 2012 and completed a nurse-administered questionnaire. A total of 615 participants who gave consent to be recontacted were sent a follow-up postal questionnaire between 2013 and 2014; 68% of them returned the full questionnaire, and 423 individuals who had pain and sleep data at both baseline and follow-up were included in the current study. The mean (SD) time between TJR and baseline nurse-administered questionnaire was 3.6 (2.1) years. The study was originally designed to study pain outcomes post-surgery. Having analyzed these data of the effects seen at year 3, we decided to perform a follow-up to provide assessments on how these variables changed over time; the meantime for the follow-up questionnaire was 5.9 years (2.3 years).

In both the initial nurse-administered questionnaire (3.6 years post-surgery) and the second postal questionnaire (5.9 years post-surgery), the participants were asked about pain, sleep and medication as follows:

#### Joint pain

The Western Ontario and McMaster Universities Osteoarthritis Index (WOMAC) pain score was used. The Likert scale 0 (no pain) to 20 (worst pain) was converted to 0 (worst pain) to 100 (no pain), and the cutoff definition of “severe” joint pain used by Wylde et al^12^ for post-TJR patients of <50 in this scale was used.

#### Neuropathic-like pain symptoms at the replaced joints (knees or hips)

The painDETECT questionnaire (PDQ) is one of several validated instruments for neuropathic pain symptoms. Scores range from 0 to 35, with >12 indicating possible neuropathic pain and ≥19 indicating likely neuropathic pain.^21^ Individuals were categorized as having possible or likely neuropathic pain based on the results of a joint-specific version of the PDQ.^21^ The validated PDQ cutoff score of >12 for possible neuropathic pain was used for the presence of neuropathic pain symptoms. This score of the PDQ has been shown to correlate with sensory abnormalities in OA patients (mechanical gain or loss, thermal gain or loss and signs of central sensitization measured by quantitative sensory testing) with a 5.6 increased risk of showing central sensitization defined as the presence of one or more of the following: hyperalgesia to mechanical stimuli (pinprick or pressure), a shift in stimulus–response curve, mechanical allodynia and/or enhanced temporal summation.^16^ The sensitivity of a PDQ score >12 for central sensitization was 50% and the specificity was 74%.^16^

#### Sleep questionnaire

The sleep subscale within the Medical Outcomes Survey was used to assess sleep quality over the past 4 weeks. The Medical Outcomes Study (MOS) Sleep Scale was developed in 1992 for the MOS, a study of patients with chronic conditions; hence, it is valid to study chronic pain. The instrument can be used to assess important aspects of sleep perceived by adults in the general population or participating in clinical studies. For example, the MOS Sleep Scale has been found to be responsive to change in the clinical trial with statistically significant improvements observed after administration of pregabalin for sleep disturbance, shortness of breath, sleep adequacy, sleep quantity and sleep problems.^22^ The survey uses Likert scales for 10 items ranging from “All of the time” to “None of the time”.^23^,^24^ We recalibrated each subscale to have better sleep corresponding to higher values and the sum of scores to 0 (worst sleep) to 100 (ideal sleep). The bottom tertile of the distribution of sleep scores (<60) was used to define disturbed sleep.

#### Medication use

Individuals were classified as taking opioids, oral nonsteroidal anti-inflammatory drugs (NSAIDs), anti-neuropathic medications and other centrally acting prescription medications that are used to treat pain and other hypnotics:

- Opioids: buprenorphine, co-codamol, codeine, co-dydramol, co-proxamol, dihydrocodeine, fentanyl, morphine, Nurofen Plus (ibuprofen and codeine phosphate), Oramorph, oxycodone, tramadol and Transtec patch (buprenorphine).
- NSAIDs: celecoxib, diclofenac, meloxicam, naproxen and piroxicam. None of the participants reported taking other prescription NSAIDs.
- Anti-neuropathic medications and other centrally acting medication used to treat pain: nefopam, gabapentin and pregabalin.
- Medications used to treat pain related to central sensitization, anxiety, depression and other psychiatric illnesses, which may have a hypnotic effect: diazepam, temazepam, sertraline, paroxetine, fluoxetine, mirtazapine, venlafaxine, amitriptyline, citalopram, dosulepin and duloxetine.

#### Binary traits

In order to compare hazard ratios (HRs) across the three main outcomes, joint pain defined by WOMAC, neuropathic joint pain symptoms defined by PDQ and disturbed sleep were converted into binary traits using the cutoffs described earlier.

### Statistical analysis

We performed a priori power calculations for the two main analyses in the study. 1) One of the analyses was the effect of sleep disturbance at baseline on predicting onset of pain at follow-up (among individuals with no severe pain at baseline). Given 288 individuals without disturbed sleep at baseline, we estimated the necessary effect size on the incidence of disturbed sleep needed between the 201 individuals without severe neuropathic pain and the 86 individuals with severe neuropathic pain. We found that an odds ratio (OR) of ≥2.1 is needed to achieve ≥80% power with *p*<0.05 and an OR of ≥1.8 is needed to achieve 60% power. 2) The other analysis was the effect of severe neuropathic pain at baseline on developing disturbed sleep at follow-up. Given 309 individuals without severe pain at baseline, we estimated the necessary effect size on incidence of severe pain needed between the 185 individuals without disturbed sleep and the 124 individuals with disturbed sleep at baseline. We found that an OR of ≥2.05 is needed to achieve ≥80% power with *p*<0.05 and an OR of ≥1.75 is needed to achieve 60% power.

Mean (SD) and n (%) were used for descriptive purposes (Table 1). Longitudinal associations between disturbed sleep (dependent variable) were assessed fitting proportional Cox regressions, and HRs were used to investigate the association between pain at baseline visit and incidence of disturbed sleep, and of disturbed sleep at baseline on incidence of joint pain (defined as WOMAC score <50), and neuropathic pain symptoms. Cox regression models were fitted including age, sex, body mass index (BMI) and use of hypnotic and analgesic medication as covariates. All statistical analyses were performed using R 3.3.1 (https://cran.r-project.org/).

## Results

A total of 423 participants, 59.0% females with mean age 69 years, participated in this study. Their descriptive characteristics are given in Table 1. At baseline, 27% of individuals had severe joint pain, 24% of them had neuropathic pain symptoms and one-third of them fell within the disturbed sleep category.

On cross-sectional analysis, both severe joint pain and neuropathic pain symptoms were associated with disturbed sleep: adjusted odds ratio (aOR) 9.32 (95% confidence interval [CI]: 5.43–16.02; *p*<0.0001) and 5.41 (95% CI: 3.27–8.94; *p*<0.0001), respectively, adjusted for age, sex, BMI and use of analgesic and hypnotic medications. Both severe pain and neuropathic pain symptoms were associated with disturbed sleep with aOR 6.1 (95% CI: 3.32–11.2; *p*<0.0001) and aOR 2.65 (95% CI: 1.49–4.70; *p*<0.0009), respectively.

The PDQ and sleep scores at baseline and follow-up depending on the presence of neuropathic pain symptoms at baseline and disturbed sleep at baseline are given in Table 2. A considerable proportion of individuals do not remain in the same category (<60% of participants with PDQ score >12 fall into this category at follow-up).

**Figure 1:**
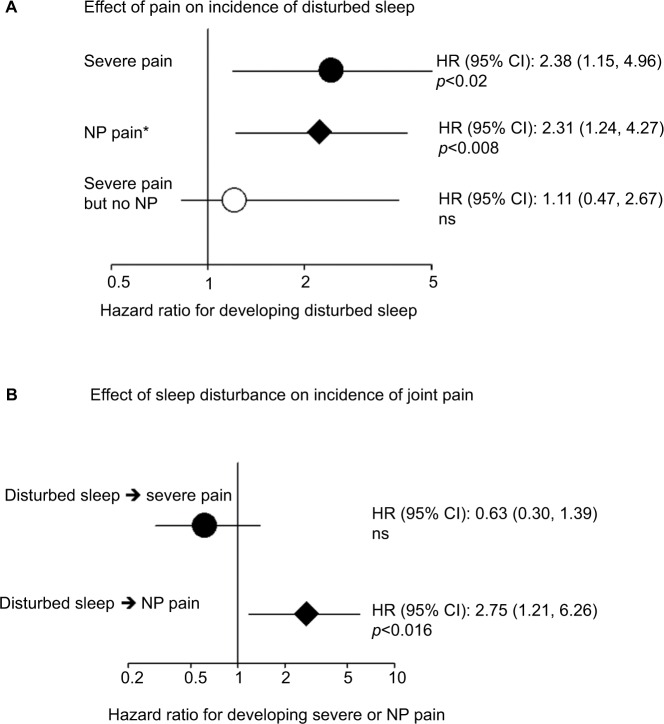
Longitudinal effect of pain on incidence of disturbed sleep and vice versa in 423 individuals post-TJR. **Notes:** (**A**) The effect of different types of pain categories (severe OA pain, NP, severe pain but excluding NP) on the development of disturbed sleep 2 years later in individuals in the top two tertiles for sleep score at baseline. Severe joint pain is defined as WOMAC score <50; presence of NP symptoms is defined as PDQ score >12. *Model includes age, sex, BMI, severe joint pain and NP. All individuals with disturbed sleep at baseline are excluded from the analysis. (**B**) Risk of developing different types of pain (NP symptoms or moderate to severe joint pain) among individuals who neither had severe joint pain (WOMAC score >50) nor NP symptoms (PDQ ≤12) at baseline depending on their sleep disturbance status (bottom tertile of the sleep scores). Individuals with severe pain or NP at baseline were excluded from the analysis. **Abbreviations:** TJR, total joint replacement; OA, osteoarthritis; NP, neuropathic pain; WOMAC, Western Ontario and McMaster Universities Osteoarthritis Index; PDQ, painDETECT questionnaire; BMI, body mass index; ns, not significant; HR, hazard ratio.

We first assessed whether pain at the time of first assessment predicted incidence of disturbed sleep at follow-up. We did this excluding from the analysis the 31% of individuals who already had disturbed sleep at baseline (ie, a sleep score <60), thus seeking to identify if there is a relationship between incident cases of disturbed sleep at follow-up and pain status at baseline. The presence of neuropathic joint pain symptoms (affecting 24.4% of individuals at baseline) predicted incidence of disturbed sleep over the 2.3-year period with a hazard ratio (HR_p_) 2.31 (95% CI: 1.24–4.27; *p*<0.008; Figure 1A). This value remained significant after adjustment for pain severity (aHR_poor_sleep_ 2.01, 95% CI: 1.00–4.10; *p*<0.05). The presence of severe joint pain was also associated with the incidence of disturbed sleep (HR_poor_sleep_ 2.38, 95% CI: 1.15–4.96; *p*<0.02). However, after excluding individuals with neuropathic pain symptoms and adjustment for use of hypnotic and analgesic medications, this was no longer significant (adjusted hazard ratio [aHR] 1.11, 95% CI: 0.47–2.67; Figure 1A), while individuals with neuropathic pain symptoms at baseline were at a high risk of developing sleep disturbance (aHR 2.31, 95% CI 1.24–4.27; *p*<0.008) and remained significant after adjustment for WOMAC pain scores.

Similarly, being in the bottom tertile of sleep scores associated with an increased risk of incident neuropathic pain symptoms after 2 years, independent of other covariates (aHR 2.75, 95% CI 1.21–6.26; *p*<0.016). However, being in the bottom tertile of sleep scores had no effect on the incidence of severe joint pain (HR 2.75, 95% CI 1.21–6.26; *p*<0.016) after adjusting for covariates and medication use (Figure 1B).

## Discussion

In this longitudinal study, we provided evidence that there is a strong temporal link between chronic joint pain and disturbed sleep in people who have undergone TJR for OA and also showed, to our knowledge for the first time, that the increased incidence of disturbed sleep is linked specifically to neuropathic pain symptoms and not merely to the presence of pain.

Our data are in agreement with previous prospective studies that showed that sleep disturbance increases the risk for new-onset chronic pain in pain-free individuals, worsens the long-term prognosis of existing headache and chronic musculoskeletal pain and adversely influences daily fluctuations in pain.^4^

The bidirectional link that we find between neuropathic joint pain and disturbed sleep is consistent with previous experimental works showing that there are common brain areas involved in both processes. Specifically, the frontal cortex and the hippocampus have been implicated both in response to sleep deprivation^25^ and in the processing of neuropathic pain^26^ in animal models. These are also areas involved in the negative effect, in particular depression and depressive symptoms, which have been shown to be important in linking sleep disturbance to pain in OA.^1^ We did not explore this additional affective dimension in the present analysis but have adjusted for the use of medications used to treat anxiety, depression and other psychiatric conditions.

Our data suggest that pain with neuropathic features and disturbed sleep augment each other in a vicious circle. Thus, addressing neuropathic pain symptoms might prevent sleep disturbances, and interventions addressing sleep disturbance should prevent the onset of neuropathic joint pain. Further randomized controlled trials are needed to test this theory, as both chronic pain and sleep disturbance are common prevalent conditions.^6^,^27^ We note several study limitations. First, our data are from a cohort recruited from secondary care and all participants had already undergone TJR surgery at the time of baseline assessment. Our findings should be generalized to other forms of joint pain in other settings with caution. Second, the sleep assessment was self-reported in questionnaires. More objective measures such as actigraphy or polysomnography might help determine whether the associations that we report are explained by alterations in sleep or in participant perceptions of sleep quality. In addition, we have classified sleep disturbance according to tertiles of questionnaire scores within our study population. Different results might have been obtained if clinical cutoffs were available defining troublesome or pathological sleep disturbance. Notwithstanding these caveats, to our knowledge, this is the first study on joint pain that demonstrates the bidirectionality of association between sleep disturbance and chronic joint pain, specifically addressing the role of neuropathic joint pain.

## Conclusion

Our study shows that neuropathic joint pain predicts incidence of poor sleep and vice versa in people with OA who have undergone TJR. These data suggest a causal bidirectional link between sleep disturbance and neuropathic joint pain but not with joint pain in general. Ameliorating sleep disturbance and joint pain has potential to improve outcomes in people after joint replacement surgery, particularly if analgesic strategies target neuropathic joint pain.

## Figures and Tables

**Table 1:** Descriptive characteristics of study participants, including proportion of patients with severe joint pain (WOMAC), disturbed sleep (MOS sleep score recalibrated to 0–100) and neuropathic joint pain (PDQ) at baseline and follow-up

Trait	Value
Participants (N)	423
Sex, female (%)	59.0
BMI (kg/m^2^), mean (SD)	29.0 (5.2)
Age (years), mean (SD)	69.7 (9.3)
TKR (%)	58.0
THR (%)	36.7
Time of baseline assessment post-surgery (years), mean (SD)	3.67 (2.45)
Time from baseline to follow-up in years, mean (SD)	2.33 (1.21)
Baseline pain and sleep	
WOMAC pain score (0–100, 100=no pain), mean (SD)	69.62 (23.47)
% of individuals with severe pain (WOMAC score <50)	27.2
PDQ score (0–35, 0=no NP symptoms), mean (SD)	7.36 (7.98)
% of individuals with neuropathic joint pain (PDQ score >12)	24.4
MOS sleep score (0–100, 100=no sleep problems), mean (SD)	69.58 (17.42)
% of individuals with sleep score <60	31.0
Follow-up pain and sleep	
WOMAC pain score (0–100) at follow-up, mean (SD)	71.66 (17.01)
% of individuals with WOMAC score <50	24.84%
PDQ score (0–35) at follow-up, mean (SD)	5.69 (7.80)
% of individuals with possible NP (PDQ>12) at follow-up	18.9%
MOS sleep score at follow-up, mean (SD)	66.89 (16.05)
% of individuals with sleep score <60	32.5%
Use of medication at baseline (%)	
Opioids	22.3
Centrally acting medication with possible hypnotic effect	8.5
Centrally acting medication used to treat pain	3.5
NSAIDs	7.6

**Abbreviations:** BMI, body mass index; NSAIDs, nonsteroidal anti-inflammatory drugs; MOS, Medical Outcomes Study; NP, neuropathic pain; PDQ, painDETECT questionnaire; THR, total hip replacement; TKR, total knee replacement; WOMAC, Western Ontario and McMaster Universities Osteoarthritis Index.

**Table 2:** Sleep and PDQ sleep scores at baseline and follow-up in four groups of post-TJR participants

NP symptoms^a^	Disturbed sleep^b^	Participants (n)	Baseline		Follow-up			
			MOS score, mean (SD)	PDQ score, mean (SD)	MOS score, mean (SD)	PDQ score, mean (SD)	NP symptoms (%)^a^	Disturbed sleep (%)^b^
0	0	275	79.69 (10.24)	3.15 (3.92)	71.62 (13.82)	2.88 (4.85)	5.1	18.2
0	1	74	50.88 (11.63)	5.04 (4.16)	61.42 (16.40)	5.77 (6.97)	17.6	48.7
1	0	43	71.86 (8.38)	18.07 (4.32)	66.67 (15.41)	11.72 (9.88)	53.5	30.2
1	1	71	47.68 (12.62)	19.85 (5.41)	55.85 (14.71)	13.31 (9.58)	55.8	69.0

**Notes:**

a NP symptoms defined from the PDQ score, 0 if PDQ score ≤12 and 1 if PDQ score >12.

b Disturbed sleep as defined from the MOS score, 0 if MOS score >60 and 1 if MOS score ≤60.

**Abbreviations:** PDQ, painDETECT questionnaire; TJR, total joint replacement; NP, neuropathic pain; MOS, Medical Outcomes Study.
